# Prognostic Model of Baseline Medications plus Neutrophil-to-lymphocyte Ratio in Patients with Advanced Non-small-cell Lung Cancer Receiving Immune Checkpoint Inhibitor plus Platinum Doublet: A Multicenter Retrospective Study

**DOI:** 10.7150/jca.80517

**Published:** 2023-03-11

**Authors:** Izumi Nasu, Masahiro Kondo, Ryuji Uozumi, Shinya Takada, Shuichi Nawata, Hirotoshi Iihara, Yohei Okumura, Masashi Takemoto, Kozo Mino, Tadanori Sasaki, Chiemi Hirose, Tohru Aomori, Rena Shimano, Ken Maeno, Satoshi Oizumi, Sojiro Kusumoto, Yasushi Ohno, Shinnosuke Ikemura, Daiya Takai, Azusa Hara, Hitoshi Kawazoe, Tomonori Nakamura

**Affiliations:** 1 Division of Pharmaceutical Care Sciences, Keio University Graduate School of Pharmaceutical Sciences, 1-5-30 Shibakoen, Minato-ku, Tokyo 105-8512, Japan; 2 Department of Pharmacy, Toranomon Hospital, 2-2-2 Toranomon, Minato-ku, Tokyo 105-8470, Japan; 3 Department of Pharmacy, Nagoya City University Hospital, 1-Kawasumi, Mizuho-cho, Miskuho-ku, Nagoya, Aichi 467-8602, Japan; 4 Department of Industrial Engineering and Economics, Tokyo Institute of Technology, 2-12-1 Ookayama, Meguro-ku, Tokyo 152-8552, Japan; 5 Department of Pharmacy, National Hospital Organization Hokkaido Cancer Center, 4-2-3-54, Kikusui, Shiroishi-ku, Sapporo 003-0804, Japan; 6 Department of Hospital Pharmaceutics, School of Pharmacy, Showa University, 1-5-8 Hatanodai, Shinagawa-ku, Tokyo 142-8666, Japan; 7 Department of Pharmacy, Gifu University Hospital, 1-1 Yanagido, Gifu, Gifu 501-1194, Japan; 8 Department of Pharmacy, Keio University Hospital, 35 Shinanomachi, Shinjuku-ku, Tokyo 160-8582, Japan; 9 Department of Respiratory Medicine, Allergy and Clinical Immunology, Nagoya City University Graduate School of Medical Sciences, 1-Kawasumi, Mizuho-cho, Mizuho-ku, Nagoya, Aichi 467-8602, Japan; 10 Department of Respiratory Medicine, National Hospital Organization Hokkaido Cancer Center, 4-2-3-54, Kikusui, Shiroishi-ku, Sapporo 003-0804, Japan; 11 Respiratory Medicine and Allergology, Showa University School of Medicine, 1-5-8 Hatanodai, Shinagawa-ku, Tokyo 142-8666, Japan; 12 Department of Cardiology and Respiratory Medicine, Gifu University Graduate School of Medicine, 1-1 Yanagido, Gifu, Gifu 501-1194, Japan; 13 Division of Pulmonary Medicine, Department of Medicine, Keio University School of Medicine, 35 Shinanomachi, Shinjuku-ku, Tokyo 160-8582, Japan; 14 Department of Respiratory Medicine, Toranomon Hospital, 2-2-2 Toranomon, Minato-ku, Tokyo 105-8470, Japan; 15 Division of Drug Development and Regulatory Science, Keio University Faculty of Pharmacy, 1-5-30 Shibakoen, Minato-ku, Tokyo 105-8512, Japan

**Keywords:** pembrolizumab, atezolizumab, immune checkpoint inhibitors, baseline medications, prognostic model, non-small-cell lung cancer

## Abstract

**Background:** Association between baseline medications plus neutrophil-to-lymphocyte ratio (NLR) and the effectiveness of immune checkpoint inhibitor (ICI) plus platinum doublet remains unknown, despite several reported prognostic models. We used real-world data to investigate whether baseline medications plus NLR predict survival outcomes in patients with advanced non-small-cell lung cancer (NSCLC) receiving ICI plus platinum doublet.

**Methods:** This multicenter, retrospective, observational study conducted in Japan between December 2018 and March 2021 used real-world data of consecutive patients with advanced NSCLC who received ICI (pembrolizumab or atezolizumab) plus platinum doublet as first-line treatment. Progression-free survival (PFS) and overall survival (OS) were estimated using the Kaplan-Meier method. The prognostic score for baseline medications plus NLR was weighted by regression β coefficients and used to categorize patients into good, intermediate, and poor prognoses groups. In addition, time-dependent receiver operating characteristic curve analyses and univariable and multivariable Cox proportional hazards models were constructed.

**Results:** Overall, 241 patients were included. Poor prognosis was significantly associated with worse PFS (hazard ratio [HR]: 1.78; 95% confidence interval [CI]: 1.08-2.94; *P* = 0.025) and OS (HR: 3.59; 95% CI: 2.05-6.28; *P* < 0.001) than good prognosis. Harrell's C-index for this prognostic model was 0.648.

**Conclusions:** Baseline medication plus NLR could predict progressively worse survival outcomes in patients with advanced NSCLC receiving ICI plus platinum doublet and could be used as a prognostic index for poor outcomes.

## Introduction

Lung cancer is one of the most commonly diagnosed cancers and the leading cause of cancer-related deaths for both sexes 1. Approximately 19.3 million new cancer cases and 10 million cancer-related deaths occurred in 2020. Lung cancer accounts for an estimated 1.8 million deaths (18%) among men and women combined. Furthermore, most patients initially diagnosed with non-small-cell lung cancer (NSCLC) are already at an advanced stage. Combination therapy comprising an immune checkpoint inhibitor (ICI) plus platinum doublet such as cisplatin and carboplatin has become the first-line treatment for advanced NSCLC 2-4. The median overall survival (OS) is approximately 5 months longer with an ICI plus platinum doublet than with placebo combination therapy 2-4, which has remarkably changed the survival outcomes of these patients.

The effect of baseline medications on the effectiveness of ICI monotherapy is considered controversial. Baseline concomitant medications include corticosteroids, antibiotics, non-steroidal anti-inflammatory drugs (NSAIDs), and proton pump inhibitors (PPIs) 5-7. However, the gut microbiome can affect ICI effectiveness. Responders and non-responders to ICI monotherapy for melanoma showed significant differences in the diversity and composition of the gut microbiome 8. Patients with a high abundance of *Faecalibacterium* in their gut microbiome had a higher density of immune cells and markers of antigen processing and presentation than those with a high abundance of *Bacteroidales*, thereby suggesting a possible mechanism through which the gut microbiome modulates anti-tumor immune responses. Another study 9 showed that the gut microbiome might modulate responses to anti-programmed cell death 1 immunotherapy. Patients with a good gut microbiome, including those with a high diversity and abundance of *Ruminococcaceae* and *Faecalibacterium*, have enhanced systemic and anti-tumor immune responses mediated by increased antigen presentation and improved effector T cell function in the tumor periphery and microenvironment. Antibiotics, NSAIDs, and PPIs can alter the gut microbiota and reduce its diversity. Additionally, the use of antibiotics and PPIs is associated with decreased OS and progression-free survival (PFS) after ICI monotherapy 10.

Systemic inflammation is associated with the prognosis of solid tumors. The neutrophil-to-lymphocyte ratio (NLR), platelet-to-lymphocyte ratio (PLR), and lymphocyte-to-monocyte ratio (LMR) are biomarkers of the general immune response to various stress stimuli 11,12. A high NLR is associated with poor OS after ICI monotherapy 11,12. Moreover, some studies reported a direct correlation between NLR and intra-tumoral levels of granulocyte myeloid-derived suppressor cells, which are closely related to neutrophils 11,12.

Importantly, these studies evaluated baseline medications and NLR or PLR separately. Buti et al. 13 and Ogiwara et al. 14 studied NLR combined with baseline medications in patients receiving ICI monotherapy. Recently, Ogura et al. 15 reported an association between immunological and nutritional markers and the outcomes of a first-line ICI plus platinum doublet treatment; however, they did not consider baseline medication usage. To the best of our knowledge, no study has investigated the association between baseline medications and NLR, PLR, or LMR and survival outcomes associated with ICI plus platinum doublet as first-line treatment. Therefore, we hypothesized that baseline medications plus NLR, PLR, or LMR could predict survival outcomes after ICI plus platinum doublet treatment in clinical practice.

This study is novel because we focused on ICI plus platinum doublet as the first-line therapy for patients with advanced NSCLC. Furthermore, several prognostic models have been reported for ICI monotherapy 13 or combination therapy 16,17. In this study, all enrolled patients had NSCLC and received ICI plus platinum doublet as first-line treatment. Moreover, we collected data of over 200 patients from six facilities across the country. Furthermore, we constructed a prognostic model that combined NLR and baseline medications in this study. The individual usefulness of NLR and baseline medications has been evaluated by Joshi et al. 17, however, there are no prognostic models that consider both NLR and baseline medications to date.

In this study, we used real-world data to investigate the prognostic model according to weighted scores in patients with advanced NSCLC treated with ICI plus platinum doublet as first-line treatment.

## Materials and Methods

### Study design

This study utilized a multicenter, retrospective, observational design. Patients' data were collected from the electronic medical records of six participating medical institutions: Toranomon Hospital, Nagoya City University Hospital, National Hospital Organization Hokkaido Cancer Center, Showa University Hospital, Gifu University Hospital, and Keio University Hospital in Japan. Data integration and analyses were performed at the Keio University Faculty of Pharmacy. This study adhered to the STROBE statement 18 and followed the methods used in previous studies 14,19,20.

The inclusion criteria were as follows: 1) consecutive patients aged ≥20 years with postoperative relapse or stage IV NSCLC and 2) patients who had received at least one course of combination therapy of ICI (pembrolizumab or atezolizumab) plus platinum doublet as first-line treatment between December 2018 and March 2021. The treatment schedule and follow-up were modified at the discretion of the clinician, according to the efficacy and/or toxicity profile of each patient.

The exclusion criteria were as follows: 1) incomplete medical records or lack of baseline laboratory data, 2) having active cancers other than advanced NSCLC, 3) comorbid autoimmune disease, 4) history of tuberculosis, 5) history of interstitial pneumonia, and 6) use of unapproved medicine in clinical trials. In addition, routine clinical follow-up of the enrolled patients was performed daily.

### Data collection

Patient data were deidentified and analyzed anonymously. Data on patients' age; sex; chemotherapy regimen; Eastern Cooperative Oncology Group performance status (ECOG PS); counts of absolute neutrophils, lymphocytes, monocytes, and platelets at baseline; date of progression and/or death at the time of initiation of the immune-platinum doublet; and baseline medications (corticosteroids [dose ≥10 mg prednisolone equivalent per day and cancer-related use], antibiotics, fibrates, statins, metformin, PPIs, and NSAIDs) were collected. Baseline medication was defined as use within 30 days of oral or intravenous administration before initiating the immune-platinum regimen 21. We calculated NLR, PLR, and LMR at baseline using routinely available blood cell counts. NLR was calculated as the absolute neutrophil count divided by the absolute lymphocyte count, PLR as the absolute platelet count divided by the absolute lymphocyte count, and LMR as the absolute lymphocyte count divided by the absolute monocyte count. The follow-up period ended on September 30, 2021.

### Endpoints

PFS was defined as the period from the date of initiating the ICI plus platinum doublet treatment to the date of progression disease (PD), whereas OS was the period from the date of initiating the ICI plus platinum doublet treatment to the date of death from any cause. Patients without documented PD or who were still alive were defined as censored to PFS and OS, respectively, on the date of the last follow-up. Computed tomography (CT) evaluation is routinely assessed and is commonly conducted after about two months from the treatment initiation. At the maintenance period, CT evaluation is assessed every around three months.

### Statistical analyses

The study endpoints were OS and PFS, which were estimated using the Kaplan-Meier method. The log-rank test was performed to compare the survival curves. Time-dependent receiver operating characteristic (ROC) curve analyses 22 and Youden's index were used to determine the optimal cutoff values for NLR associated with OS. In the ROC curve analyses, a higher area under the curve (AUC) indicated better predictive ability. We calculated prognostic scores using regression β coefficients via a univariable analysis, as reported in previous studies 7,13,14. Univariable and multivariable Cox proportional hazards models were used to examine the associations between the groups based on prognostic scores and survival outcomes. In sensitivity analysis, potential explanatory variables regarding patients' background, including age (10-year intervals) and ECOG PS (2 vs. 0-1), were included in the multivariable model as covariates. The explanatory variables were included in multivariable analysis by the enter method. These explanatory variables were determined based on our clinical judgment. Additionally, we calculated another prognostic scores using regression β coefficients via a multivariable analysis.

Discrimination was assessed by concordance probability estimates using Harrell's C-index 23. All statistical analyses were performed using SAS (version 9.4; SAS Institute, Cary, NC, USA) and SPSS Statistics (version 25; IBM, Armonk, NY, USA). All *P*-values were two-sided, and statistical significance was set at a *P*-value of <0.05.

### Ethics statement

The study protocol was approved by the Ethics Committee of Toranomon Hospital (approval number: 2225), Nagoya City University Hospital (approval number: 60-21-0074), National Hospital Organization Hokkaido Cancer Center (approval number: 03-15), Showa University Hospital (approval number: 3503), Gifu University Hospital (approval number: 2021-0188), Keio University Hospital (approval number: 20210049), and Keio University Faculty of Pharmacy (approval number: 220518-4). This study was conducted in accordance with the Declaration of Helsinki and the Ethical Guidelines for Medical and Health Research involving Human Subjects by the Ministry of Education, Culture, Sports, Science, and Technology, and the Ministry of Health, Labour and Welfare of Japan. Written informed consent for participation in this study was waived in accordance with the national legislation and institutional requirements.

## Results

### Patient characteristics

**Figure 1:**
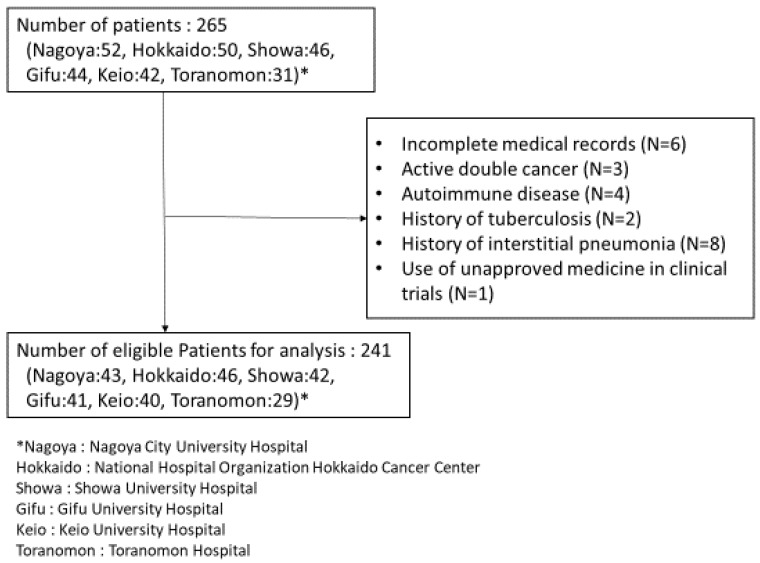
Flowchart of the patient enrollment process

The patient enrollment flowchart is shown in **Figure 1**. Of the 265 patients initially screened, 24 were excluded from the analysis. Data of the remaining 241 patients were finally evaluated in this study. Patient characteristics are listed in **Table 1**. The median patient age was 68 years (interquartile range [IQR]: 61-72 years). Overall, 211 (87.6%) patients were in good condition, with an ECOG PS score of 0-1. In total, 195 (80.9%) and 46 (19.1%) patients received pembrolizumab and atezolizumab, respectively. The most frequently used ICI plus platinum doublet treatment was pembrolizumab + carboplatin + pemetrexed in 119 patients (49.4%), and the most frequently used baseline medications were PPIs and NSAIDs in 87 (36.1%) and 83 (34.4%) patients, respectively.

### Endpoints

The median PFS and OS were 0.73 and 2.04 years, respectively. In the univariable analysis, the concomitant use of PPIs and NSAIDs resulted in significantly decreased OS (**Table 2**). In contrast, no significant association was observed between other concomitant medications and OS.

**Figure 2:**
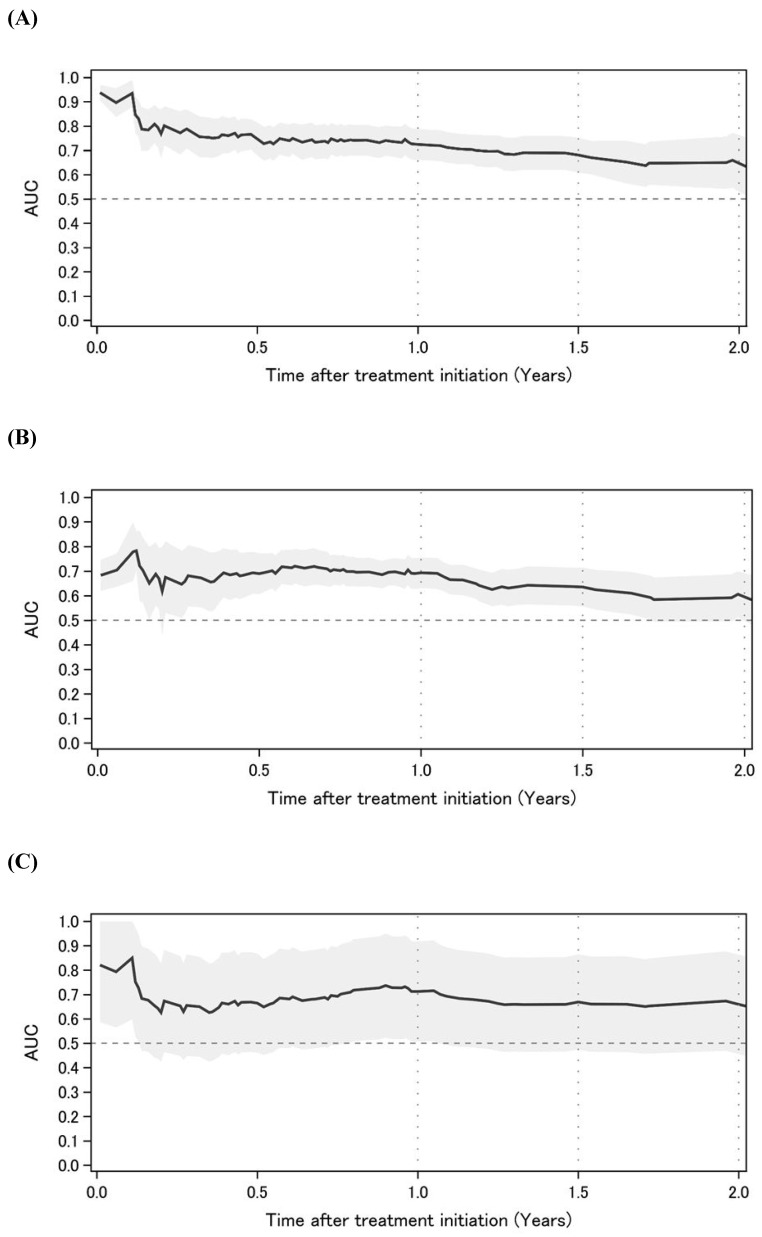
Time-dependent AUC of NLR, PLR, and LMR** (A)** NLR. **(B)** PLR. **(C)** LMR. Abbreviations: AUC: area under the curve; NLR: neutrophil-to-lymphocyte ratio; PLR: platelet-to-lymphocyte ratio; LMR: lymphocyte-to-monocyte ratio.

**Figure 3:**
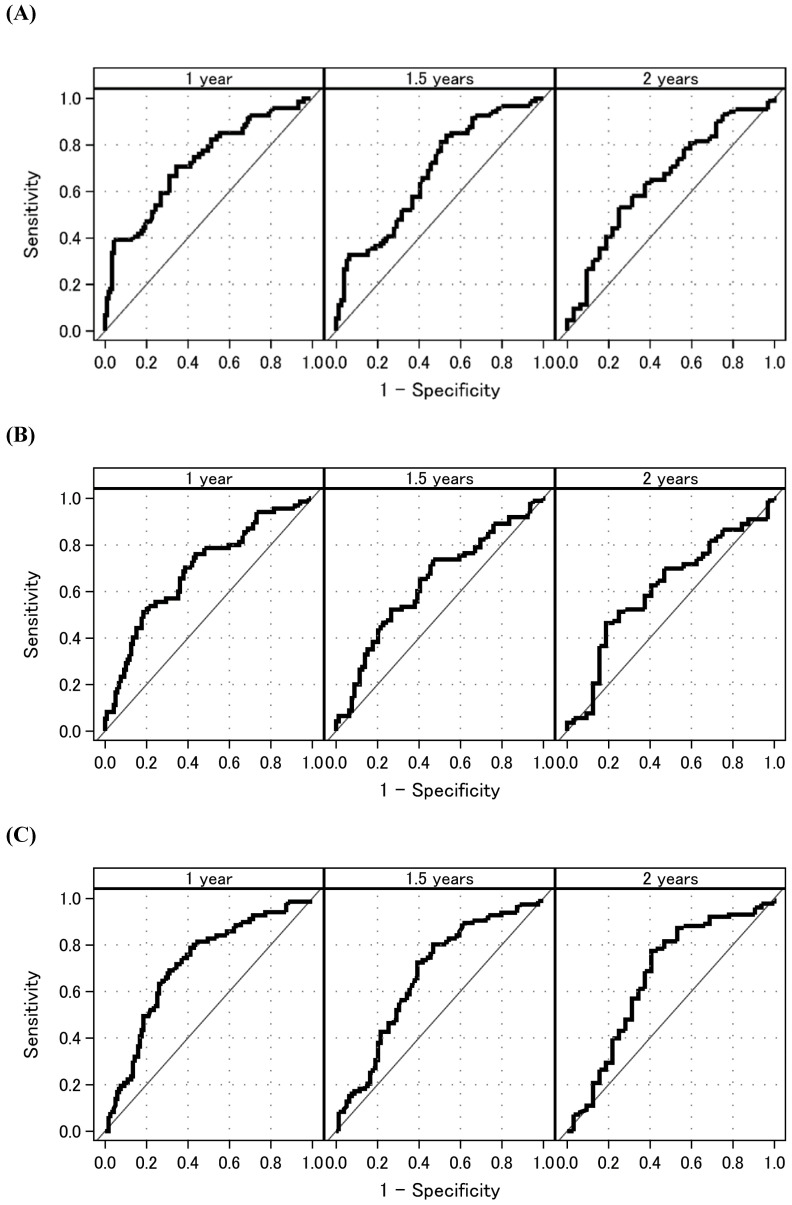
Time-dependent ROC curve analyses to determine the optimal cutoff values for NLR, PLR, and LMR to predict overall survival** (A)** NLR. **(B)** PLR. **(C)** LMR. Time-dependent ROC curves at 1, 1.5, and 2 years. Abbreviations: ROC: receiver operating characteristic; NLR: neutrophil-to-lymphocyte ratio; PLR: platelet-to-lymphocyte ratio; LMR: lymphocyte-to-monocyte ratio.

**Figure 2** shows the time-dependent AUCs of NLR, PLR, and LMR. The time-dependent AUC of NLR was slightly higher than that of PLR and LMR. However, the time-dependent AUC of NLR remained higher than 0.6 over time. Subsequently, time-dependent ROC curve analyses to determine the optimal cutoff values for NLR, PLR, and LMR to predict OS are shown in **Figure 3**. At 1, 1.5, and 2 years after initial treatment, the optimal NLR cutoff values were 4.2, 3.2, and 4.9 (**Figure 3A**); the optimal PLR cutoff values were 296, 209, and 298 (**Figure 3B**); and the optimal LMR cutoff values were 2.4, 3.1, and 3.2 (**Figure 3C**), respectively. In particular, the AUC (95% confidence interval [CI]) for NLR was 0.725 (0.661-0.788), 0.681 (0.609-0.772), and 0.653 (0.539-0.767) at 1, 1.5, and 2 years after initial treatment, respectively (**Figure 3A**). However, the AUCs for PLR and LMR were lower than that for NLR (**Figures 3B and 3C**). Therefore, we selected NLR for subsequent analysis. Given the clinical importance and statistical analysis, we chose the NLR cutoff value of 4.2 at 1 year after initial treatment.

### Prognostic factors and scoring

**Figure 4:**
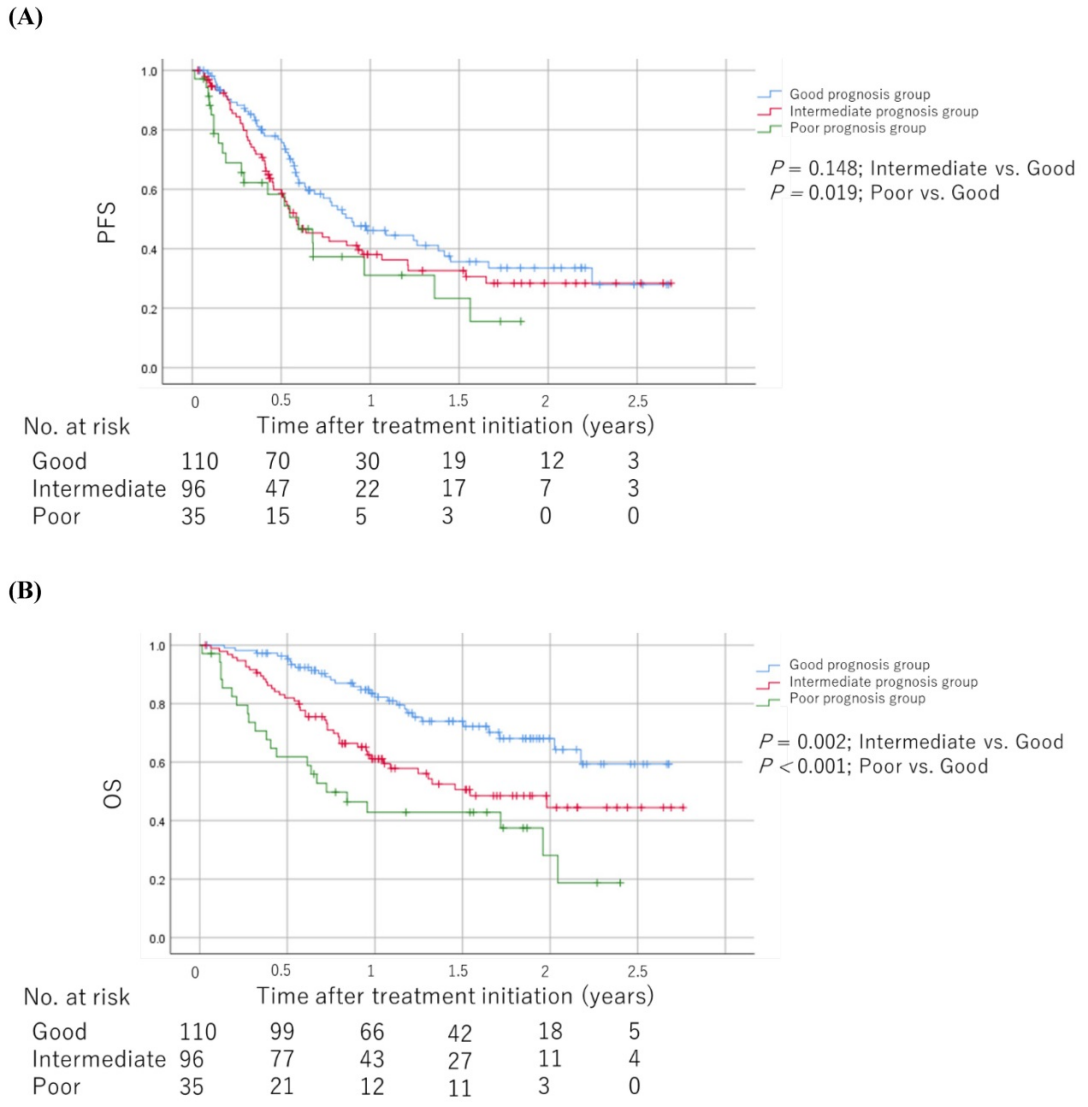
Kaplan-Meier survival curves for PFS and OS among the groups scored based on regression β coefficients via a univariable analysis **(A)** PFS. **(B)** OS. The log-rank test was used to compare survival curves. Abbreviations: PFS: progression-free survival; OS: overall survival.

As shown in **Table 2**, we developed prognostic scores with baseline medications plus NLR based on regression β coefficients via a univariable analysis as follows: patients with an NLR ≥4.2 were assigned 2 points, whereas 1 point each was allotted for PPIs, NSAIDs, and corticosteroid use. We allotted 0 points each for an NLR <4.2 and no PPIs, NSAIDs, and corticosteroid use. Therefore, each patient was assigned a score ranging from 0 to 5. We categorized patients into the good (score 0-1), intermediate (score 2-3), and poor (score 4-5) prognosis groups based on their scores. The Kaplan-Meier survival curves for PFS and OS among the groups are shown in **Figure 4**. The poor prognosis group had a significantly worse PFS than the good prognosis group (log-rank test; *P* = 0.019). In addition, the poor and intermediate prognosis groups had significantly worse OS than the good prognosis group (log-rank test; *P* < 0.001 and *P* = 0.002, respectively). The univariable Cox proportional hazards model showed that the poor prognosis group had significantly worse PFS and OS than the good and intermediate prognosis groups (hazard ratio [HR]: 1.78; 95% CI: 1.08-2.94; *P* = 0.025 and HR: 3.59; 95% CI: 2.05-6.28; *P* < 0.001, respectively). Sensitivity analysis using the multivariable Cox proportional hazards model for OS revealed consistent results (**Table 3**). The HRs for OS were 3.03 (95% CI: 1.66-5.52, *P* < 0.001) and 2.30 (95% CI: 1.39-3.79, *P* = 0.001) in the poor and intermediate prognosis groups, respectively. Applying the computed score to this population, Harrell's C-index for OS was 0.643.

**Figure 5:**
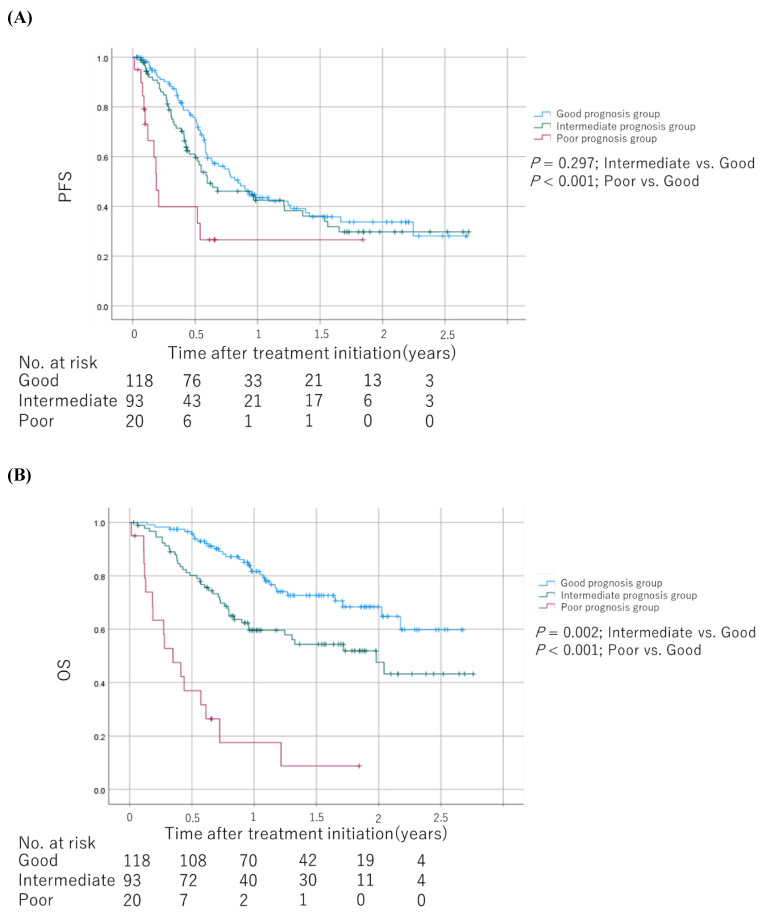
Kaplan-Meier survival curves for PFS and OS among the groups scored based on regression β coefficients via a multivariable analysis **(A)** PFS. **(B)** OS. The log-rank test was used to compare survival curves. Abbreviations: PFS: progression-free survival; OS: overall survival.

We calculated another prognostic scores based on regression β coefficients via a multivariable analysis as follows: patients with ECOG PS ≥2 and NLR ≥4.2 were assigned 2 points and 1 point, respectively (**Table 2**). Therefore, each patient was assigned a score ranging from 0 to 3. We categorized patients into the good (score 0), intermediate (score 1), and poor (score 2-3) prognosis groups based on their scores. The Kaplan-Meier survival curves for PFS and OS among the groups are shown in **Figure 5**. The poor and intermediate prognosis groups had a significantly worse OS than the good prognosis group (log-rank test; *P* < 0.001 and *P* = 0.002, respectively).

## Discussion

Our study findings suggest that baseline medications plus NLR can progressively worsen survival outcomes in patients with advanced NSCLC receiving ICI plus platinum doublet as first-line treatment in clinical practice. The poor and intermediate prognosis groups had lower OS than the good prognosis group. To the best of our knowledge, this is the first study to investigate baseline medications plus NLR as a prognostic factor in patients treated with the ICI plus platinum doublet for advanced NSCLC using real-world data. Additionally, weighted scoring combined with baseline medications and NLR can predict prognosis. Several prognostic models have also been reported for ICI monotherapy. The mechanism underlying the association between a higher NLR and survival outcomes remains unclear. However, ICIs interrupt immune suppression and activate CD8+ T-lymphocytes in the tumor microenvironment. Similarly, the mechanism underlying the association between baseline medications and survival outcomes remains unclear, except for corticosteroids. The diversity of the gut microbiota might influence the effectiveness of ICIs because of their association with immune status 24. The present study showed an association between a higher NLR and survival outcomes and between baseline medications and poor survival outcomes. Integrating the time-dependent AUC of NLR showed a good prognostic factor that maintained a high predictive ability over time. The lymphocyte count is immutable and impossible to modify before ICI treatment. In contrast, baseline medications can be stopped or changed to a different medication. New agents with different mechanisms of action from ICIs or those that do not increase lymphocyte counts are needed for patients with poor prognosis. Furthermore, some agents may enhance the effects of ICIs by altering the gut microbiota 25. Therefore, the prognosis can be improved by concomitant oral administration of this prebiotics with ICIs.

This study had a gap of over 1 year between OS and PFS. A previous study reported that ICIs have a carry-over effect 26, and there is sometimes a pseudo-progression before ICIs prove to be effective 27. This may be because patients diagnosed with the progressive disease based on the pseudo-progression who stop ICIs still present a continuous effect of ICIs, leading to longer OS.

Regarding baseline medications, Buti et al. 14 reported significant differences in survival between the use of corticosteroids and antibiotics. Compared to NLR, the contribution of NSAIDs and PPIs are less; thus, we assigned 1 point for NSAIDs and PPIs and 2 points for NLR ≥4.2. In the cohort training study by Buti et al. 7, corticosteroids are strongly associated with the worsened outcome ICI treatment. In our study, 10 among 241 cases used corticosteroids, whereas Buti et al. reported 50 of 217 such cases. It is obvious clinically that corticosteroids are the first class of medication identified to be significantly related to worse clinical outcomes among patients treated with ICIs. We considered the detection power in our study to be low because of a small number of cases of corticosteroid use. However, whether corticosteroids should be used for palliative or non-palliative purposes for cancer-related conditions remains controversial 28-30. While corticosteroids could reduce the efficacy of ICIs, a reduction in efficacy has not been seen 6. In the present study, 10 (4.1%) patients were treated with concomitant corticosteroids, whereas in other studies, 93 (14.3%) of 650 patients received more than 10 mg of prednisone equivalents in both palliative and non-palliative settings. However, this study was conducted only in Japan, and antibiotic use might differ from that in other countries and in the extent of change in microbiota.

In this study, the settings of patients' backgrounds were different from those in previous studies because ICI plus platinum doublet for advanced NSCLC is a new regimen as first-line treatment. Buti et al. 13 reported that in 950 patients with advanced NSCLC who received pembrolizumab monotherapy as first-line treatment, a drug-based prognostic score of baseline medications showed a predictive ability for survival outcomes. Each concomitant drug was assigned a different score, and patients were categorized into three groups using regression β coefficients. Ogiwara et al. 14 reported that in 259 patients with advanced NSCLC who received nivolumab and pembrolizumab monotherapy as first- and later-line treatments, respectively; the prognostic score of baseline medications plus NLR had a higher predictive value than that reported by Buti et al. In the present study, Harrell's C-index was comparable to that reported by Buti et al. and Ogiwara et al. 13,14.

Buti et al. constructed a prognostic model based on regression β coefficients via a univariable analysis 13. We also developed a prognostic model based on regression β coefficients via a univariable analysis and clinical decision. Additionally, we developed another prognostic model consisting of ECOG PS ≥2 and NLR ≥4.2 based on regression β coefficients via a multivariable analysis. The poor and intermediate prognosis groups had lower OS than the good prognosis group, as in the previous prognostic model.

The present study has several strengths. First, it was a multicenter study of six participating institutions in Japan, including cancer centers, university hospitals, and community hospitals. Therefore, our data may be generalizable to similar populations in the clinical setting. Second, we focused on the ICI plus platinum doublet treatment for patients with advanced NSCLC because of administering a new regimen as first-line treatment. Third, we performed time-dependent ROC curve and sensitivity analyses using the multivariable Cox proportional hazards model, which resulted in consistent results. These analyses increased the robustness of our results and are the novel features of our study.

This study has several limitations. First, this was a retrospective observational study. Therefore, information bias could not be excluded. There is also the possibility of missing clinical and drug history data. While we did not determine the tumor proportion score (TPS) using programmed death-ligand 1 expression data, it can be assumed that this did not affect the impact of baseline medications plus NLR on survival outcomes, and there was no need to determine the TPS when using ICI plus platinum doublet for advanced NSCLC. Additionally, oncogenic driver mutation data were not collected. When oncogenic driver mutations are present, tyrosine kinase inhibitors should be used as first-line treatment. We assumed that the histological type, such as squamous cell carcinoma and adenocarcinoma, is not a prognostic factor in patients receiving ICIs; therefore, data on the histologic type were not collected. However, besides the unmeasured confounders mentioned above, other baseline medications and second-line treatment were not adjusted in the multivariable analyses, which is a major limitation of our study because controlling for these could have affected the results. Second, the sample size was relatively smaller than that in previous studies that developed a drug-based prognostic score for the first time 7,14. The present study only included a training cohort. Further validation cohorts are warranted to confirm the clinical application of this prognostic model. Third, we did not collect data on immune-related adverse events (irAEs), although the development of irAEs was positively correlated with survival outcomes in previous reports 31-33. In contrast, Miura et al. 6 reported that baseline medications were not significantly associated with the onset of irAEs in 300 Japanese patients with advanced NSCLC treated with nivolumab or pembrolizumab monotherapy. In addition, the longer the patients survive, the higher the incidence of irAEs. Fourth, we did not consider the patients with no confirmed PD were treated as censors on the date of their last CT evaluation due to a clinical practice setting. There may be an overestimation of PFS. However, we believe that OS analyses were robust. Finally, this study mainly included a Japanese population. If the microbiota is associated with the effectiveness of ICI plus platinum doublet treatment, the outcome might change based on the country, the trend of medication use, food culture, and race, which are associated with microbiota diversity. Overall, the findings should be validated in a large-scale multicenter study with a large sample of patients treated with various baseline medications that affect the microbiota.

## Conclusions

Baseline medications plus NLR at baseline could predict shorter survival in patients treated with ICI plus platinum doublet as first-line treatment for advanced NSCLC. In addition, the findings from this multicenter nationwide study conducted in Japan can be translated to a Japanese population.

## Figures and Tables

**Table 1:** Baseline patient characteristics

Characteristic	Total (*n* = 241)
Age, years	
Median (IQR)	68 (61-72)
Sex, *n* (%)	
Male	177 (73.4)
Female	64 (26.6)
ECOG PS, *n* (%)	
0	98 (40.7)
1	113 (46.9)
2	20 (8.3)
Unknown	10 (4.1)
ICI plus platinum doublet, *n* (%)	
Pembrolizumab + CBDCA + PEM	119 (49.4)
Pembrolizumab + CBDCA + nab-PTX	52 (21.6)
Pembrolizumab + CDDP + PEM	18 (7.5)
Pembrolizumab + CBDCA + PTX ± bevacizumab	6 (2.5)
Atezolizumab + CBDCA + PTX ± bevacizumab	25 (10.4)
Atezolizumab + CBDCA + nab-PTX	19 (7.9)
Atezolizumab + CBDCA + PEM	1 (0.4)
Atezolizumab + CDDP + PEM	1 (0.4)
Baseline concomitant medications, *n* (%)	
Corticosteroids^a^	10 (4.1)
Antibiotics	59 (24.5)
PPIs	87 (36.1)
NSAIDs	83 (34.4)
Metformin	14 (5.8)
Fibrates	8 (3.3)
Statins	46 (19.1)
Baseline peripheral blood counts (cells/mm^3^), median (IQR)	
Absolute neutrophil count	4846 (3694-6699)
Absolute lymphocyte count	1303 (889-1687)
Platelet count	275000 (225500-351000)
Absolute monocyte count	483 (338-656)
NLR, median (IQR)	3.89 (2.70-5.87)
PLR, median (IQR)	230.8 (158.4-329.2)
LMR, median (IQR)	2.52 (1.78-3.78)

^a^ Dose ≥10 mg prednisolone equivalent per day and cancer-related use Abbreviations: IQR: interquartile range; ECOG PS: Eastern Cooperative Oncology Group performance status; ICI: immune checkpoint inhibitor; CBDCA: carboplatin; PEM: pemetrexed; nab-PTX: albumin-binding paclitaxel; CDDP: cisplatin; PTX: paclitaxel; PPIs: proton pump inhibitors; NSAIDs: non-steroidal anti-inflammatory drugs; NLR: neutrophil-to-lymphocyte ratio; PLR: platelet-to-lymphocyte ratio; LMR: lymphocyte-to-monocyte ratio

**Table 2:** Regression β coefficients from univariable and multivariable analyses for overall survival

	Univariable analysis				Multivariable analysis		
Variables	Regression β coefficient	Crude HR (95% CI)	*P*-value		Regression β coefficient	Adjusted HR (95% CI)	*P*-value
NLR ≥4.2	0.939	2.56 (1.68-3.90)	<0.001		0.865	2.38 (1.50-3.77)	<0.001
PPIs	0.597	1.82 (1.21-2.73)	0.004		0.318	1.38 (0.86-2.19)	0.181
NSAIDs	0.452	1.57 (1.04-2.37)	0.032		0.125	1.13 (0.69-1.86)	0.622
Corticosteroids	0.211	1.23 (0.45-3.37)	0.681		0.110	1.12 (0.34-3.68)	0.856
Age (10-year interval)	0.120	1.13 (0.90-1.41)	0.293		0.069	1.01 (0.98-1.38)	0.586
ECOG PS ≥2	1.926	6.86 (3.92-12.01)	<0.001		1.683	5.38 (2.95-9.82)	<0.001

Ten patients with unknown ECOG PS were excluded from this analysis. Abbreviations: HR: hazard ratio; CI: confidence interval; NLR: neutrophil-to-lymphocyte ratio; PPIs: proton pump inhibitors; NSAIDs: non-steroidal anti-inflammatory drugs; ECOG PS: Eastern Cooperative Oncology Group performance status.

**Table 3:** Multivariable Cox proportional hazards model for overall survival

Variables		No.	Event	Censored	Adjusted HR (95% CI)	*P*-value
Group	Poor	33	21	12	3.03 (1.66-5.52)	<0.001
	Intermediate	92	40	52	2.30 (1.39-3.79)	0.001
	Good	106	26	80	1	
Age (10-year interval)		-	-	-	1.09 (0.85-1.39)	0.489
ECOG PS	2	20	16	4	6.01 (3.25-11.10)	<0.001
	0-1	211	71	140	1	

Ten patients with unknown ECOG PS were excluded from this analysis. Abbreviations: HR: hazard ratio; CI: confidence interval; ECOG PS: Eastern Cooperative Oncology Group performance status.

## Abbreviations

CBDCA: carboplatin
CDDP: cisplatin
CI: confidence interval
ECOG PS: Eastern Cooperative Oncology Group performance status
ICI: immune checkpoint inhibitor
irAE: immune-related adverse event
IQR: interquartile range
LMR: lymphocyte-to-monocyte ratio
nab-PTX: albumin-binding paclitaxel
NLR: neutrophil-to-lymphocyte ratio
NSAIDs: non-steroidal anti-inflammatory drugs
NSCLC: non-small-cell lung cancer
OS: overall survival
PD: progression disease
PEM: pemetrexed
PFS: progression-free survival
PLR: platelet-to-lymphocyte ratio
PPIs: proton pump inhibitors
PTX: paclitaxel
ROC: receiver operating characteristic
